# Reduced Milk Production, Economic Losses, and Risk Factors Associated to Subclinical Hypocalcemia in Holstein Friesian × Zebu Crossbreed Cows in North-West Ethiopia

**DOI:** 10.3389/fvets.2022.771889

**Published:** 2022-03-29

**Authors:** Sefinew Alemu Mekonnen, Zegeye Alelgn, Seid Saudik, Wassie Molla, Tsegaw Fentie, Wudu Temesgen Jemberu

**Affiliations:** ^1^Department of Veterinary Epidemiology and Public Health, College of Veterinary Medicine and Animal Sciences, University of Gondar, Gondar, Ethiopia; ^2^Wag Hemra Zone Livestock and Fishery Development Office, Sekota, Ethiopia

**Keywords:** crossbreed cows, economic loss, Ethiopia, subclinical hypocalcemia, risk factor

## Abstract

Hypocalcemia is a metabolic disorder that occurs when calcium leaves the blood to support milk production or for the growth of the fetus faster than calcium can be put back into the blood. Subclinical hypocalcemia (SCH) refers to clinically normal cows but with concentrations of calcium lower than measured in normal cows. A study was conducted to estimate economic losses and to identify risk factors associated with SCH in Holstein Friesian × Zebu crossbreed Cows in North-West Ethiopia. A total of 221 lactating dairy cows obtained from 81 dairy farms were selected and serum samples were collected and analyzed to estimate the level of calcium in the serum using an automated electrolyte analyzer (Roch, UVL Japan, 2014). Forty-seven cows obtained from 12 farms were grouped into two: cows with SCH and cows without SCH and milk yield was measured for eight consecutive days to estimate milk production loss associated with SCH. Prevalence was estimated and univariable and multivariable logistic regression analyses were used to identify determinants of SCH. The prevalence of SCH was 20.3% (51/221, 90% CI = 18.6%−28.1%). Grazing on pasture, membership to dairy cooperative, feeding above the cows' requirement (based on farmers' opinion), and previous experience of metritis reduced the odds of SCH (*P* < 0.05). The average economic losses associated with reduced milk production due to SCH were 11.25 Ethiopian Birr (ETB) (1 ETB = 0.023 US Dollar) and 3,026.25 ETB (69.6 US Dollar) per cow per lactation, respectively. The main findings of the study have shown that SCH was a problem in crossbreed dairy cows in Northwestern Ethiopia and identified few risk factors that could be targeted to mitigate the problem. Actually, in this study, the economic cost of SCH was significant and needs attention in the dairy extension and dairy health training programs.

## Introduction

With 7.2 million dairy cows, Ethiopia is endowed with the largest livestock population in Africa (1). However, the annual growth rate in milk production cannot satisfy the milk demand of the growing population (2). Challenges that the dairy sector in Ethiopia are facing such as the low genetic potential of indigenous breeds of cows, shortage of feed, livestock diseases, poor management practice, and lack of an organized marketing system (3). To balance the milk supply to the milk demand, besides increasing the number of dairy cows, increasing milk production per animal is an additional way to go through cross-breeding. The Ethiopian government is taking action to improve the genetic potential of indigenous cows for higher milk yield through crossbreeding with Holstein Friesian semen and the number of cows with Holstein Friesian blood levels is increasing currently. However, higher milk yield in high-producing dairy cows leads to an increase in the metabolic demand of the cow (4) which increases the incidence of metabolic disorders (5).

Hypocalcemia is one of the most important metabolic disorders in dairy cows worldwide (6, 7) that reaches peak incidence between calving and peak of lactation. Hypocalcemia occurs when calcium in the blood leaves to support milk production faster than calcium that can be put back into the blood from the diet, skeletal calcium stores, and renal conservation of calcium. Subclinical hypocalcemia (SCH) refers to when concentrations of calcium are lower than measured in normal cows but have no clinical symptoms of hypocalcemia (8–10). However, there is inconsistency regarding what concentration of total calcium in serum should be used as the cut-point. The most common cut-point cited is the concentration of calcium <2 mmol/l in the serum (9). SCH, as compared to clinical hypocalcemia, is considered to occur at high prevalence in the postpartum cow has an unrecognized problem that may contribute to the development of most postpartum production diseases (9) that are responsible for severe economic losses in terms of heavy reduction in milk yield, impaired fertility, and increased the risk of other metabolic diseases such as ketosis (11). There is also a strong relation between a low level of calcium and impaired immune function leading to infectious disease (4). A low level of calcium reduced muscular tone in teat end sphincters in hypocalcemic cows (12). A low level of calcium reduces a cow's innate immunity as a result it increases the chance of the cow being infected by mastitis-causing bacteria (4, 13). In Ethiopia, hypocalcemia is a common problem in dairy cattle (14–17). Although the severity of the disease is greater in clinical cases, subclinical cases are also important because they are far more frequent and are responsible for large economic losses. However, they cannot be easily diagnosed (4, 18) and are not well recognized by many farmers (9).

Although many articles have been published on hypocalcemia (6–8, 19), specific information on the epidemiology and economic impact of hypocalcemia in smallholder farms with low milk yields are limited. To get a better estimate of the costs of milk fever and SCH specifically for the situation in smallholder farms with low milk yields, more studies are needed. The identification of risk factors associated with the occurrence of hypocalcemia can help to improve programs for the prevention and control of milk fever in dairy herds. Worldwide, many studies have been conducted on the management of milk fever, and many risk factors were found to be associated with the occurrence of milk fever (19–21). In Ethiopia, there is likely an increased prevalence of SCH, as the Holstein Friesian blood level was found to be positively associated with the prevalence of hypocalcemia (14). Therefore, specific attention to risk factors for hypocalcemia in this country is valuable. Estimates of costs of hypocalcemia specifically the cost of SCH may motivate farmers to take preventive measures. By calculating the costs of SCH, awareness of the financial consequences of hypocalcemia can be created. Consequently, the possibility of financial returns from preventing hypocalcemia can be illustrated which is a first step to prevent hypocalcemia and an important part of farmers' motivation to implement control.

In this study, we collected specific parameters needed to calculate costs of SCH per cow through questionnaires and the number of cows with SCH by testing serum samples collected from individual cows. We aimed to estimate the prevalence of SCH, to identify factors associated with SCH, and to estimate average economic losses associated with reduced milk production due to SCH in urban and peri-urban dairy farms with Holstein Friesian × Zebu crossbreed lactating dairy cows in Northwestern Ethiopia.

## Materials and Methods

### Study Area

This study was conducted in and around Gondar town in Central Gondar zone, in Amhara National Regional State. Gondar town is located between 12°36' North latitude and 27°2' East longitude. The annual mean minimum and maximum temperature vary between 12.3–17.7°C and 22–30°C, respectively, with an annual average temperature of 19.7°C (1).

### Study Herds and Cows

All cows used in the study were Holstein Friesian × Zebu crossbreed cows. The majority of herds were kept indoors and were hand-fed. Hay, wheat bran, crop residue, and brewery byproduct were used to feed the cows. Cows were milked by hand two times a day early in the morning and late in the afternoon by stripping (milking by thumb and index finger), squeezing (milking by five fingers), or both, depending on the habit of the farmer and the size of the teats.

This study consists of two separate studies: prevalence and risk factors study and milk production losses and economics study. In the prevalence and risk factors study, data were collected from 81 dairy farms to gain insight into the prevalence and risk factors associated with SCH. The 81 dairy farms were obtained by random selection from the extensive list of dairy herds in Gondar town. In the milk production losses and economics study, data were collected on 12 farms to evaluate the effect of SCH on milk production losses. For this later study, dairy farms that had relatively “large herds,” dairy farms with a herd size > 6 cows, with “the same” breed and management were selected based on their willingness to participate in the study.

### Study Design

#### Selection of Cows for Prevalence and Risk Factor Study

A cross-sectional study was conducted to estimate the prevalence of SCH and to identify risk factors associated with SCH. As there was no formal list of dairy farms, a list of dairy farms was recorded by using information from veterinary clinics, artificial inseminators' records, and “Lame Bora” dairy cooperative aiming to represent the population of dairy herds in Gondar town. Based on random selection, 81 dairy farms were selected from the list. In the selected dairy farms, all lactating cows were taken if the number of lactating cows in a herd was ≤ 4. If the number of lactating cows within a herd was > 4, four cows were randomly selected. In that way, 221 lactating cows were participated in this study.

After proper restraining of the cows, about 5 ml of blood sample was collected from the jugular vein into 10 ml plain vacutainer tubes. Blood samples were stored in a vertical rack and transported to the laboratory within 5 h of sampling. The samples were properly labeled and stored in the icebox and transported to the University of Gondar Veterinary public health laboratory for serum separation. The serum was separated and transferred into Eppendorf tubes, Hamburg, Germany, labeled and kept at −20°C until the serum calcium was measured. Serum calcium was measured by using an automated serum electrolyte machine that uses atomic absorption spectrophotometer (HumaLyte Plus^5^ Ion-Selective Electrolyte Analyzer) and Roche-986, UVL, Japan, 2014. Cows with a serum calcium of <2 mmol/l were considered subclinically hypocalcemic otherwise free of SCH (9).

#### Design of the Questionnaire and Study Variables

For the risk factor study, a questionnaire aiming to collect data was prepared to address two main issues. First part of the questionnaire was addressed characteristics of the farmer and that of the herd. The second part of the questionnaire contained questions regarding each cow enrolled in the study such as, Holstein Friesian blood level (HFBL), parity, experience of the cow to common hypocalcemia-related health problems (lameness, metritis, retained placenta, uterine prolapse, abnormal vaginal discharge, dystocia, abortion, milk fever, clinical ketosis, and mastitis), and calving date were included. All questionnaires mainly contained closed questions. The original questionnaire was prepared in English before it was translated to the farmers' local language (Amharic) and subsequently translated back into English by an external translator to check consistency. The final questionnaire was administered by face-to-face interviews. The English version of the questionnaire is available (Supplementary Material 1). All the 221 lactating crossbreed cows from selected herds were examined for body condition score by visual inspection. Body condition was scored in three levels: poor, good, and very good. The information on the levels of HFBL was estimated based on artificial inseminator records and history from the farmers.

Data were collected on herd-level and cow-level variables. Six herd-level variables referring to dairy farmers' personal characteristics and herd characteristics were studied (Table 1). Five of the variables were recorded in two levels. One variable, the level of feeding, was recorded in three levels according to the farmers' opinion.

**Table 1:** Herd-level variables potentially related to subclinical hypocalcemia in dairy farms of North-West Ethiopia as included in the questionnaire.

**Group variables**	**Variable**	**level**
Farmer's characteristics	Membership of a dairy cooperative	No/Yes
	Receiving expert advice about hypocalcemia	No/Yes
	Herd size	≤5 animals, 6–9 animals, and >9 animals
Herd characteristics	Level of feeding	Below the requirement
		Meet the requirement
		Above the requirement
	Use of pasture grazing	No/Yes
	Deworming experience	No/Yes

Sixteen cow-level variables were selected as potential risk factors and consequences of SCH were included in the questionnaire (Table 2). Average lactation length, parity, and body condition score were recorded into three categories the rest 13 variables were in two levels.

**Table 2:** Cow-level variables potentially related to subclinical hypocalcemia in dairy farms of North-West Ethiopia as included in the questionnaire.

**Variables**	**Level**
Exotic breed blood level	≤50, >50
Parity	1, 2, >3
Lameness	No/Yes
Metritis	No/Yes
Retained placenta	No/Yes
Uterine prolapse	No/Yes
Abnormal vaginal discharge	No/Yes
Abortion	No/Yes
Dystocia	No/Yes
Mastitis	No/Yes
Milk fever	No/Yes
Clinical ketosis	No/Yes
Average lactation length in months	1–7, 8–9, ≥10
Average milk yield per day	≤9, >9
Pregnant status	No/Yes
Body condition score	Poor, Good, Very Good

#### Milk Production Losses

Forty-seven cows obtained from 12 dairy farms were used to estimate the reduction in milk yield due to SCH. Each cow was matched with another cow in breed, parity, and milk yield within a farm; if a cow had no mach in a farm that cow did not participate in the study. To control the effect of breed and management on milk yield, dairy farms that had “the same” breed and management were used and serum separated from blood samples as mentioned earlier. Serum calcium was measured by using an automated serum electrolyte machine that uses atomic absorption spectrophotometer (HumaLyte Plus^5^ Ion-Selective Electrolyte Analyzer) and Roche-986, UVL, Japan, 2014. Cows with a serum calcium of <2 mmol/l were considered subclinically hypocalcemic otherwise free of SCH (9). The 47 cows were grouped into two: cows with SCH (*N* = 8) and cows free of SCH (*N* = 39) based on the level of calcium in the serum. As there was no standardized milk yield recording scheme operating on dairy farms in the study area, the 47 cows were hand milked, and the milk was put separately in a bucket. The half a day milk yield was measured using calibrated milk measuring jar over 8 days period in both groups of cows. Although cows were milked two times daily, only the late-afternoon milking (half a day milking) was measured. The average daily milk production loss due to SCH was estimated by subtracting the average daily milk yield in cows with SCH from the average daily milk yield of cows without SCH over the eight-day period. Finally, this amount was multiplied by two (doubled) (Equation 1) to get the daily milk yield estimate.

### Statistical Analysis

The data collected from 81 farms that were used to test for potential herd-level and cow-level risk factors were analyzed as follows. The univariable screening was done using mixed logistic regression models with a random herd effect. Correlations between pairs of independent variables were evaluated using the Spearman rank correlations. If two variables had a correlation coefficient of ≥ |0.7|, only one of the variables was included in the further multivariable analysis. Both herd-level and cow-level risk factors that were statistically significant at *P* < 0.25 in the univariate analyses were tested starting from the most significant variable by removing one variable at a time in the same multivariate multilevel logistic regression models using backward reduction. Variables in multivariable models with *P* < 0.1 from the Wald test were retained. All the two-way interactions between variables in the final multivariable models were tested, but no significant interactions were found. Confounding was checked during the model building process by evaluating the change in the beta estimate of other variables when a variable was removed from the models. If this change in beta estimate was > 30%, the variable was considered a confounder. Dystocia and retained placenta were strongly correlated with metritis (*r* = 0.78); therefore, these two variables were excluded from the analyses. The data analysis was performed using Stata release14 (StataCorp LLC, USA).

### Calculation of Economic Losses

Economic losses of SCH were estimated after estimating the average daily milk production losses of SCH by subtracting the average daily milk yield in cows with SCH from the average daily milk yield of cows without SCH over the 8 days period. Costs of SCH were calculated based on the average daily milk production loss due to SCH per cow in combination with information obtained from the questionnaire. As farmers did not have the information on the average daily milk production loss, we used the half-a-day milk yield measurement over the 8 days to estimate the average daily milk production losses associated with SCH. Economic losses were determined as costs per cow per lactation. To estimate these costs, we made the following assumptions:

1. Because the parity of cows was generally unknown to the farmers, all SCH animals were considered to occur in the same average parity.
2. Because dairy farmers did not have any cow records, lactation length was uncertain. Therefore, the costs of SCH per lactating cow were estimated at an average lactation length of 269 days.
3. The milk production level was considered constant throughout lactation.
4. Milk yield in the morning milking was considered equivalent to milk yield in the afternoon milking.

Cows with blood total calcium threshold <2 mmol/l were considered subclinical hypocalcemic (9).


(1) ADMLCSCH =(∑i=18DMYCcSCH NCcSCH×8−​∑i=18DMYCSCH​NCcSCH×8)∗2

Where:

*ADML*_*CSCH*_ = Average daily milk production losses per cow associated with SCH based on the half a day milk yield measurement over 8 days

*DMY*_*CȼSCH*_ = Total half a day milk yield in liter in cows without SCH over an eight-day period

*N*_*CȼSCH*_ = Number of cows without SCH

*DMY*_*CSCH*_ = Total half a day milk yield in liter in cows with SCH over an eight-day period

*N*_*CSCH*_ = Number of cows with SCH

Economic losses as a result of the reduction in milk yield associated with SCH per cow per day was estimated as


(2) $$\begin{array}{l} ELCD_{SCH}=ADML_{CSCH}\;*\;P_{M} \end{array}$$

Where:

*ELCD*_*SCH*_ = Economic losses associated with SCH per cow per day

*ADML*_*CSCH*_ = Average daily milk production losses per cow associated with SCH based on the half a day milk yield measurement over 8 days

*P*_*M*_ = The average price of milk per liter in Ethiopian Birr (ETB) during the study period

Economic losses per cow per lactation as a result of the reduction in milk yield associated with SCH was estimated as


(3) $$\begin{array}{l} ELCL_{SCH}=ELCD_{SCH}\;*\;L_{L} \end{array}$$

Where:

*ELCL*_*SuM*_ = Economic losses per cow per lactation associated with SCH

*ELCD*_*SCH*_ = Economic losses associated with SCH per cow per day

*L*_*L*_ = Lactation length

Milk prices used in the calculations were derived from a questionnaire survey that was conducted among the owners of the participating farms. Data used to calculate costs of SCH are available in Supplementary Material 2.

## Results

### Descriptive Statistics

The prevalence of SCH was 20.3% (51/221, 90% CI = 18.6–28.1%). There was a great variation in the prevalence of SCH among the different groups of cows. The highest prevalence of SCH was estimated in cows with poor body condition (40%) while the lowest was in cows with metritis (4.17%). Based on the univariate logistic regression analysis, six of the variables were significantly associated with SCH (*p* < 0.25) (Table 3).

**Table 3:** Descriptive statistics and univariate associations (*P* < 0.025) between potential risk factors and subclinical hypocalcemia (*N* = 221) with a random herd effect in dairy farms of North-West Ethiopia.

**Variable**	**Level**	**Number of animals examined**	**SCH^a^ (%)**	**OR^b^ (75% CI^c^)**	***P* value**
Herd size	≤5 animals	36	9 (25)	Ref.^d^	
	6–9 animals	89	25 (28)	1.17 (0.69–1.97)	0.72
	> 9 animals	96	17 (18)	0.65 (0.38–1.11)	0.35
Membership to dairy cooperative	No	113	31 (27.43)	Ref.	
	Yes	108	20 (18.52)	0.60 (0.41–0.87)	0.12
Pasture grazing	No	185	46 (24.86)	Ref.	
	Yes	36	5 (13.89)	0.49 (0.27–0.89)	0.16
Opinion on the level of feeding	Below	53	19 (35.85)	Ref.	
	Meet	150	29 (19.33)	0.43 (0.29–0.64)	0.012
	Above	18	3 (16.67)	0.358 (0.16–0.79)	0.14
Deworming status	No	47	9 (19.15)	Ref.	
	Yes	174	42 (24.14)	1.34 (0.84–2.16)	0.47
Average lactation length in (months)	1–7	52	11 (21.15)	Ref.	
	8–9	68	15 (22.06)	1.05 (0.63–1.77)	0.91
	≥10	101	25 (24.75)	1.23 (0.76–1.97)	0.62
Advice about milk fever	No	45	8 (17.78)	Ref.	
	Yes	176	43 (24.43)	1.49 (0.91–2.45)	0.35
Exotic breed blood level	≤50	72	14 (19.44)	Ref.	
	>50	149	37 (24.83)	1.37 (0.91–2.05)	0.37
Parity	1 time	51	11 (21.57)	Ref.	
	2 times	56	11 (19.64)	0.89 (0.51–1.54)	0.81
	≥3 times	114	29 (25.44)	1.24 (0.78–1.97)	0.59
Lameness	No	196	44 (22.45)	Ref.	
	Yes	25	7 (28)	1.34 (0.78–2.33)	0.54
Metritis	No	197	50 (25.38)	Ref.	
	Yes	24	1 (4.17)	0.13 (0.04–0.42)	0.05
Retained placenta	No	182	39 (21.43)	Ref.	
	Yes	39	12 (30.77)	1.63 (1.04–2.56)	0.21
Uterine prolapse	No	213	50 (23.47)	Ref.	
	Yes	8	1 (12.5)	0.47 (0.13–1.62)	0.48
Abnormal vaginal discharge	No	177	42 (23.73)	Ref.	
	Yes	44	9 (20.45)	0.83 (0.51–1.33)	0.65
Dystocia	No	194	48 (24.74)	Ref.	
	Yes	27	3 (11.11)	0.38 (0.18–0.79)	0.13
Abortion	No	180	43 (23.89)	Ref.	
	Yes	41	8 (19.51)	0.77 (0.47–1.27)	0.55
Milk fever	No	211	49 (23.22)	Ref.	
	Yes	10	2 (20)	0.83 (0.33–2.09)	0.81
Clinical ketosis	No	212	49 (23.11)	Ref.	
	Yes	9	2 (22.22)	0.95 (0.37–2.44)	0.95
Mastitis	No	168	41 (24.4)	Ref.	
	Yes	53	10 (18.87)	0.72 (0.46–1.13)	0.41
Milk yield per cow(liter)	≤9	111	24 (21.62)	Ref.	
	>9	110	27 (24.55)	1.18 (0.82–1.70)	0.61
Pregnant status	No	132	29 (21.97)	Ref.	
	Yes	89	22 (24.72)	1.17 (0.80–1.69)	0.63
Body condition score	Poor	5	2 (40)	Ref.	
	Good	108	23 (21.29)	0.41 (0.14–1.20)	0.34
	Very good	108	26 (24.07)	0.48 (0.16–1.40)	0.43

a *Subclinical hypocalcemia*.

b *Odds ratio*.

c *Confidence interval*.

d *Reference category*.

### Factors Associated With Subclinical Hypocalcemia

Four variables were excluded from the final multivariate model. Grazing on pasture, membership to dairy cooperative, feeding meeting and above the cows' requirement (based on farmers' opinion), and previous experience of metritis reduced the odds of SCH (Table 4).

**Table 4:** Final multivariate mixed logistic regression models describing the association (*P* < 0.05) between risk factors and subclinical hypocalcemia based on data on 221 cows of 81 dairy farms in North-West Ethiopia and modeling a random herd effect.

**Variable**	**Level**	**OR^a^ (90% CI^b^)**	***P* value**
Grazing on pasture	No	Ref.^c^	
	Yes	0.30 (0.12–0.74)	0.03
Opinion on level of feeding requirement	Below requirement	Ref.	
	Meet the requirement	0.37 (0.19–0.68)	0.01
	Above the requirement	0.19 (0.06–0.65)	0.03
Metritis	No	Ref.	
	Yes	0.12 (0.02–0.67)	0.04
Membership to dairy farm association	No	Ref.	
	Yes	0.54 (0.31–0.96)	0.08

a *Odds ratio*.

b *Confidence interval*.

c *Reference category*.

### Costs of Subclinical Hypocalcemia

Cows with and without SCH had an average daily milk yield of 9.45 and 9.90 L, respectively, as estimated by half-a-day milk yield measurement of 8 days. The minimum, average, and maximum values of the most important input items and economic loss associated with SCH are shown in Table 5. Daily milk yield of cows and milk price varied largely between farms.

**Table 5:** Descriptive statistics of model input parameters and economic loss associated with subclinical hypocalcemia based on half-a-day milk yield measured on 8 days in 47 Holstein Friesian × Zebu crossbreed lactating cows on 12 dairy farms in North-West Ethiopia.

**Parameter**	**Minimum**	**Average**	**Maximum**
Daily milk yield of cows without SCH^a^ (in L)	1	9.90	26
Daily milk yield of cows with SCH (in L)	1	9.45	25
Average lactation length	30	269	450
Price of milk per liter during the study period	23	25	27
Total Calcium ion (mmol/L)	0.82	1.78	2.52
Reduction in milk production per day in SCH cows (in L)	0	0.45	1
Economic loss per cow per day in ETB^b^	0	11.25	27
Economic loss per cow per lactation in ETB	0	3,026.25	12,150

a *Subclinical hypocalcemia*.

b *Ethiopian Birr (1 ETB = 0.023 US Dollar)*.

## Discussion

### Prevalence of Subclinical Hypocalcemia

The objectives of this study were to estimate prevalence, identify factors associated with SCH, and estimate average economic losses associated with reduced milk production as a result of SCH in urban and periurban dairy farms keeping Holstein Friesian × Zebu crossbreed lactating dairy cows in Northwestern Ethiopia.

The prevalence of SCH was 20.3%. We collected samples regardless of the stages of lactation although the prevalence of SCH is expected to be high at the early stages of lactation (22). Therefore, the prevalence of SCH in the current would be much higher if the sampling were done at the early stage of calving, particularly in the first 48 h (9). To our knowledge, there are no studies on SCH in Ethiopia and other developing countries even though SCH is more common in dairy farms (23, 24) and is responsible for large economic losses than the clinical form (24). Higher milk yield in high-producing dairy cows leads to increased metabolic demand on the cow (4), resulting in an increased incidence of metabolic disorders (5). Therefore, it was not surprising to find the prevalence of 20.3% of SCH in the study farms which keep improved, high producing animals. Researchers from many parts of the world described that hypocalcemia (both clinical and subclinical) occur most commonly at the early stage of lactation (22, 25). For instance, Reinhardt et al. (9) from the United States of America reported a prevalence of SCH varying from 25 to 54%. However, the report was based on data at early lactation and there is a difference in production system and breed of cows that made comparison impossible.

### Risk Factors of Subclinical Hypocalcemia

Grazing on pasture, level of feeding, metritis, and membership to dairy farmers' cooperative were associated with SCH. It is not surprising to find lower odds of SCH in herds grazing on pasture compared to herds that did not graze on pasture as a low calcium diet promotes efficient resorption of calcium from bone and absorption from the small intestine. Cows that were, according to the farmers, fed above their requirement had lower odds of SCH. This might be due to the fact that dairy farmers may feed dietary calcium that was enough to meet their cows' requirements. Feeding low calcium diets in the late dry period activate calcium homeostatic mechanisms, and the cow is thus capable of absorbing calcium more efficiently from the intestine and drawing calcium from the bone around the time of calving (20). However, there is no availability of formulated rations sufficiently low in calcium in the Northwestern Ethiopian market to implement the low-calcium principle. Alternatively, these farmers can prevent SCH by feeding their cows calcium-rich rations 3–4 days before parturition, vitamin D supplementation, reducing the dietary cation-anion difference, and magnesium supplementation in the late gestation period (4).

In this study, metritis was found to be associated with reduced odds of SCH. Hypocalcemia impairs immune function and muscle contraction (18). In such a situation, metritis is more prone to occur (26). Martinez et al. demonstrated that cows with low calcium at least once between 0 and 3 days in milk had 4.5-fold increased odds of metritis (26). In a recent study in multiparous cows, Rodríguez et al. (27) also found greater odds of SCH in cows with metritis. Contradictory to many reports, in this study, cows with metritis have lower odds of SCH. This could be due to unobserved confounding in our analysis.

Membership to dairy cooperatives reduced the odds of SCH. Farms registered in dairy cooperatives had a better opportunity of getting training about different health problems of dairy cows. It can also be explained in relation to the experience in dairy farming. Farmers register in dairy cooperatives when their experience in dairy management is enough to decide to continue in dairy farming. In our study, we used serum electrolyte analysis which is not a perfect test to identify the true status of SCH, with both the imperfect sensitivity and specificity. Therefore, the strength of the associations between independent and dependent variables has probably been underestimated suggesting that some risk factors could not be identified.

### Costs of Subclinical Hypocalcemia

People first have to consider they have a problem before they will be motivated to solve that problem (28). Accordingly, one of the first prerequisites for dairy farmers to implement measures to control SCH is creating awareness that SCH is an economic problem. It is, however, less likely that Northwestern Ethiopian dairy farmers are aware of SCH as costly, and even may not know SCH as a problem at all. To our knowledge, there has never been a study that estimated the economic losses associated with SCH and milk fever in Ethiopia. By estimating the economic impact of hypocalcemia, it may be possible to motivate the prevention of hypocalcemia in Ethiopia.

The average economic losses associated with SCH were 11.25 ETB (1 ETB = 0.023 US Dollar) and 3,026.25 ETB per cow per day and per lactation, respectively. Although we tried to base the estimate of the costs of SCH as much as possible on actual data, it was not possible to obtain all the needed data from this study. Therefore, we had to make assumptions, for instance, it was assumed that milk production level was considered constant throughout lactation. Because of this assumption, there could be potentially under or overestimation of the economic impacts of SCH. Moreover, converting the 8 days measurement of SCH toward a whole year would impact the precision of the estimate of the economic losses associated with SCH. As there is no previous study that estimated economic losses associated with SCH in Ethiopia, it was not possible to compare the estimate in this study with the economic losses associated with SCH in other previous studies. However, as most of the input values in this study were based on data collected directly from cows and dairy farmers, the study gives valuable insight into the economic impacts associated with SCH.

It is less likely that Ethiopian dairy farmers are aware of the existence of SCH because there is no routine measurement of calcium level or any other parameters to monitor SCH. In a situation where farmers are neither aware of the prevalence nor of the economic consequences of SCH, it is unlikely that farmers will make efforts to prevent milk SCH. Therefore, counseling dairy farmers about the impact of SCH and the potential profit of milk fever control measures is important to motivate and change in behavior of dairy farmers to improve health of dairy cows. Moreover, dairy farmers should be informed about the importance of feeding cows with diets low in calcium in the late dry period and/or calcium-rich rations 3–4 days before parturition, vitamin D supplementation, and magnesium supplementation in the late gestation period in preventing SCH. Even though it was not possible to compare with previous results, the prevalence of SCH was high in Northwestern Ethiopian dairy farms. Few risk factors were associated with SCH indicating the importance of a better understanding of factors that predispose to deficiency of calcium in Holstein Friesian × Zebu crossbreed cows in North-West Ethiopia. Milk production losses estimated in this study were moderate. However, the economic estimate had shown that Northwestern Ethiopian dairy farmers are losing larger income due to SCH. The results in this study may be useful to aware other smallholder dairy farmers with low milk yields in Ethiopia and other countries with similar circumstances.

## Data Availability Statement

The original contributions presented in the study are included in the article/Supplementary Material, further inquiries can be directed to the corresponding author.

## Ethics Statement

Ethical review and approval was not required for the study on human participants in accordance with the local legislation and institutional requirements. The patients/participants provided their written informed consent to participate in this study.

## Author Contributions

SM and WJ conceived the study and did the statistical analysis, and economic models. SM, ZA, and SS collected the data. ZA and SS collected the samples and did the laboratory work. SM, TF, WM, and WJ supervised the project administration. ZA and SM prepared the original draft. All the authors revised and edited the manuscript and approved the final version of the manuscript for submission to a journal.

## Funding

This study was financially supported by the University of Gondar, Ethiopia with grant Ref. No. CVMAS 13/1103/03/11.

## Conflict of Interest

The authors declare that the research was conducted in the absence of any commercial or financial relationships that could be construed as a potential conflict of interest.

## Publisher's Note

All claims expressed in this article are solely those of the authors and do not necessarily represent those of their affiliated organizations, or those of the publisher, the editors and the reviewers. Any product that may be evaluated in this article, or claim that may be made by its manufacturer, is not guaranteed or endorsed by the publisher.
