# Supplementary material for: Reduced Milk Production, Economic Losses, and Risk Factors Associated to Subclinical Hypocalcemia in Holstein Friesian × Zebu Crossbreed Cows in North-West Ethiopia
Source: Front Vet Sci. 2022 Mar 29;9:771889. doi: 10.3389/fvets.2022.771889 (PMC9001979; doi:10.3389/fvets.2022.771889)
Supplement: Supplementary file 1 [file Table_1.DOC]

**Questionnaire on Economic Losses and Risk Factors of Subclinical Hypocalcemia**

Date__________

Dairy farm number: __________

Name of the dairy farm _______________________________

Adress of the farm : Zone _________________ Kebele ________________________

Owners’ demographic profile:

Gender: Male Female

Level of education__________________________________

1. When did you start dairy farming? ___________________________
2. What was the average herd size during the last year? _________________
3. What was the number of cows? Lactating? ____________ Non lactating?______________
4. Are you member of a dairy farmers association? Yes No
5. Do you milk colostrums? Yes No
6. Do your cows graze on pasture? Yes No
7. How often you supply water? Two times a day Other (specify) ____________
8. Do you supply concentrate? Yes No

If yes, how often? Consistently infrequently

The amount of concentrate you are supplying per cow per day? ______________Kg

1. What do you think about the level of feeding of your cows?

Below the requirement they get the requirement above the requirement

1. Do you use deworming to your cows? Yes No

If yes, how often? ______________

1. What is the average daily milk yield of the cows in your farm in liter per day)? ________ liters
2. What is the average lactation length of the cows in your farm (in months)? _________Months
3. What is the average value of a liter of milk? _______________ETB/liter

**Specific questions about Hypocalcemia**

1. Do you know whether milk fever exists? Yes No
2. Do you get advice about milk fever control? Yes No
3. Are you cautious to prevent cows from milk fever? Yes No

What measures are you taking? **_______________________**

1. Do you believe that milk fever is a preventable disease? Yes No
2. Did any of the cows in your farm experience milk fever during the last one year?

Yes No If yes, how many of them? _________________________

1. Do you treat cows when affected by milk fever? Yes No
2. How many of cows affected by milk fever in the last year were: treated? ______ not treated? _______
3. How much do you pay in average for Vet labour to treatment a case of milk fever? _________ETB.
4. What are the costs of the drug used to treat one case of milk fever? _____________ETB.
5. Do you think a cow loses milk production when she suffers from milk fever? Yes No

If yes, how much do you think the reduction in milk yield?__ liters/day. For how many days? --------

1. Did you remove/cull cows because of milk fever? Yes No
2. How many cows you removed/culled because of milk fever in the last two years? ___________
3. What much was the average price of a cow culled due to milk fever? ____________ETB.
4. How much it cost you in average to buy a healthy cow to replace a culled cow? __________ETB

**Cow-level questions**

1. Name Breed _____________________
2. Breed of the cow? Local _______ Cross ________
3. Holstein Friesian blood level < 25% 25-50% >50%
4. Age of the cow ________________
5. Parity of the cow ________________
6. Experience of the cow for common dairy health problems

| **Dairy health problem** | **Experience of the cow** | |
| --- | --- | --- |
|  | **Yes** | **No** |
| Lameness |  |  |
| Metritis |  |  |
| Retained placenta |  |  |
| Uterine prolapse |  |  |
| Abnormal Vaginal discharge |  |  |
| Dystocia |  |  |
| Abortion |  |  |
| Milk fever |  |  |
| Ketosis |  |  |
| Mastitis |  |  |

1. Length of dry period before the last calving ______________
2. Days in milk (Calving date) ________________
3. What is the average daily milk yield of the cow?_____ liters/day
4. Pregnancy status Pregnant Not preganant
5. Posture Normal Aberrated
6. Body condition score Poor Good Very good
